# *DsLCYB* Directionally Modulated β-Carotene of the Green Alga *Dunaliella salina* under Red Light Stress

**DOI:** 10.4014/jmb.2208.08044

**Published:** 2022-10-31

**Authors:** Yanhong Lan, Yao Song, Yihan Guo, Dairong Qiao, Yi Cao, Hui Xu

**Affiliations:** Microbiology and Metabolic Engineering Key Laboratory of Sichuan Province, College of Life Sciences, Sichuan University, Chengdu 610065, P.R. China

**Keywords:** β-Carotene, lycopene β cyclase, carotenoid, *Dunaliella salina*, transgene

## Abstract

Carotenoids, which are natural pigments found abundantly in wide-ranging species, have diverse functions and high industrial potential. The carotenoid biosynthesis pathway is very complex and has multiple branches, while the accumulation of certain metabolites often affects other metabolites in this pathway. The *DsLCYB* gene that encodes lycopene cyclase was selected in this study to evaluate β-carotene production and the accumulation of β-carotene in the alga *Dunaliella salina*. Compared with the wild type, the transgenic algal species overexpressed the *DsLCYB* gene, resulting in a significant enhancement of the total carotenoid content, with the total amount reaching 8.46 mg/g for an increase of up to 1.26-fold. Interestingly, the production of α-carotene in the transformant was not significantly reduced. This result indicated that the regulation of *DsLCYB* on the metabolic flux distribution of carotenoid biosynthesis is directional. Moreover, the effects of different light-quality conditions on β-carotene production in *D. salina* strains were investigated. The results showed that the carotenoid components of β-carotene and β-cryptoxanthin were 1.8-fold and 1.23-fold higher than that in the wild type under red light stress, respectively. This suggests that the accumulation of β-carotene under red light conditions is potentially more profitable.

## Introduction

‘Carotenoids’ is a general term for a class of valuable, natural fat-soluble pigments that are distributed widely in bacteria, fungi, algae [1-4], and photosynthetic plants. In humans, however, carotenoids can only be obtained from food. Studies to elucidate the regulation of carotenoid composition in model species and accumulate target components are critical because carotenoids have important physiological functions, including an antioxidant role, immune regulation, and prevention of cardiovascular disease, certain types of cancer, eye-related diseases, and light-induced skin damage [5].

Carotenoids are divided into two major classes: xanthophylls, which contain oxygen, and carotenes, which mainly consist of hydrocarbons and no oxygen. β-Carotene, the most important and effective vitamin A precursor among the carotenes, plays a vital role in human health, protecting against age-related degenerative diseases, cardiovascular disease, vitamin A deficiency (VAD), and certain cancers [6, 7]. β-Carotene is catalyzed directly by β-carotene hydroxylase (BCH) to generate β-cryptoxanthin, an antioxidant that may help prevent free radical damage to biomolecules including lipids, proteins, and nucleic acids [8, 9]. Studies in animal models and humans have also shown that β-cryptoxanthin derived from food has better in vitro bioavailability than α-carotene and β-carotene. Zeaxanthin, a direct product of cryptoxanthin, is a new type of oil-soluble natural pigment, which often coexists with lutein, β-carotene, and β-cryptoxanthin in nature to form a carotenoid mixture. Zeaxanthin prevents the oxidation of lipids and vitamins in food and prolongs the preservation period of food, making it an ideal natural food preservative [10, 11]. Recognition of the importance of the rich variety and functional diversity of carotenoids has resulted in increased demand and focus on how to improve the production of natural carotenoids.

The accumulation of certain metabolites often affects the composition of the carotenoid biosynthesis pathway. The cyclization reaction of lycopene converts lycopene into α-carotene and β-carotene and is an essential reaction for the production of β-carotene, which has important physiological functions in algae [12, 13]. The lycopene β cyclase (LCYB) gene is related directly to the production of β-carotene, and therefore it is necessary to study this enzyme. It has been reported that modification of the LCYB gene affects the accumulation of its metabolites and the response to abiotic stress in some photosynthetic plants. For example, in transgenic sweet potatoes, *IbLCYB2* was shown to enhance abiotic stress tolerance and carotenoid content, including α-carotene, β-carotene, lutein, β-cryptoxanthin, and zeaxanthin [14]. Heterologous overexpression of *DcLCYB1* in carrots was reported to increase *Nicotiana tabacum* fitness through positive regulation of the carotenoid, gibberellin, and chlorophyll pathways, with higher β-carotene levels reflecting increased xanthophyll production [15]. The accumulation of β-carotene in potato tubers was also shown to be regulated by *StLCYB* expression, while in tobacco, overexpression of *NtLCYB* led to increased accumulation of β-carotene, violaxanthin, lutein, and neoxanthin [16]. The LCYB gene has been cloned from *Dunaliella* species [17], although its functional characterization in the carotenoid metabolic pathway has not been studied.

Carotenoids are an important photosynthetic pigment related to the photosynthetic system. Their synthesis is a highly complex process and is stimulated by conditions that hinder cell division such as light, high temperatures, low nitrogen concentrations, or any event that produces high concentrations of intracellular reactive oxygen species (ROS) [18-20]. Among these factors, light promotes the accumulation of carotenoids in photosynthetic organisms, and is divided into different qualities and intensities according to wavelength and density [21]. In lettuce (*Lactuca sativa* ‘Banchu Red Fire’ and ‘Red Cross’), the carotenoid content was shown to be increased significantly under blue light (BL) stress [22, 23]. Huang *et al*. also reported that light-regulated changes in carotenoid metabolism during grapefruit ripening, with treatment of red light (RL) transmission causing 62%higher total carotenoid content than natural light [24]. Fu *et al*. showed that shading promoted the accumulation of carotenoids in tea, especially α-carotenoids and β-carotene [25]. A small number of studies have investigated the effect of different light qualities on carotenoid levels in photosynthetic organisms. In this regard, the unicellular alga *D. salina* is an ideal model organism for studying the production of natural carotenoids, as it has the advantages of rapid growth and no cell wall structure. In this study, we functionally identified and overexpressed *DsLCYB* to investigate whether it enhanced the accumulation of carotenoids and altered carotenoid composition in *D. salina* under light stress.

## Materials and Methods

### Materials and Growth Conditions

*D. salina* (also named FACHB-435) was obtained from the Institute of Hydrobiology, Chinese Academy of Sciences (China), and was passaged and preserved by our laboratory [26]. *D. salina* was cultivated in liquid DeWalnés medium containing 1.5 M NaCl [26, 27] and then grown with shaking [28] under different light conditions including black light (BL), 80 μmol (photons)/m^2^/s, red light (RL), 80 μmol/m^2^/s, and white light (WL, 80 μmol/m^2^/s) using SuperFlux LED, at 26 ± 1°C and 16/8 h (light/dark). When the number of algal cells under RL was about 2×10^6^ cells/ml, this was considered as the initial sampling time point (0 h). According to previous experimental results in our laboratory, the pigment content begins to show significant differences under different light conditions at this time. The algal cells were collected after 12, 24, 48, and 72 h, with 3 biological replicates obtained at each experimental time point. All the samples were frozen in liquid nitrogen immediately and stored at -80°C.

### Gene Expression Analysis

The total RNA of each sample was extracted using the TRIzol reagent (Invitrogen, USA) [29]. Reverse transcription was performed with the PrimeScript™ RT Reagent Kit with gDNA Eraser (TaKaRa, Japan), while RT-qPCR was performed on a CFX96 Touch Real-Time PCR System (Bio-Rad, USA) with SYBR Premix Ex Taq™ (Takara) using TB Green™ Premix Ex Taq™ II (TaKaRa). The specific primers are listed in Table S1, with the β-tubulin gene used as an internal reference. Three independent biological and technical replicates of each sample were subjected to RT-qPCR analysis.

### Sequence and Phylogenetic Analysis of *DsLCYB*

The 30 identified LCYB protein sequences from the 30 species were aligned using two multiple alignment methods (ClustalW and MUSCLE). The phylogenetic tree was constructed with MEGA7 software [30]. Bootstrap (BS) values were inferred from 1000 replicates. The radial tree was drawn using FigTree v1.3.1 (http://tree.bio.ed.ac.uk/software/figtree), and the MEME online program (http://meme.nbcr.net/meme/intro.htmL) was used to identify conserved motifs in the full-length identified LCYB proteins [31]. To visualize the conserved motifs, the sequences were analyzed with WEB LOGO programs (http://weblogo.berkeley.edu).

### Heterologous Expression of DsLCYB in *E. coli* BL21 (DE3) Cells

The full-length CDS of *DsLCYB* was amplified by PCR (primers shown in Table S2) using synthetic cDNA as the template, and then inserted into the pET-28a (+) plasmid (P0023, Miaoling Bioscience & Technology Co., Ltd., China) and further transformed into *E. coli* BL21 (DE3) competent cells. The transformed *E. coli*-pET-28a(+)-*DsLCYB* cells were grown separately at 37°C in LB medium containing kanamycin until the OD_600_ reached 0.5~0.6. Isopropyl β-D-thiogalactoside (IPTG) was then added to a final concentration of 0.1 mM. Cultivation was continued at 16°C and 180 rpm/min for 24 h. The cells were harvested and re-suspended in 20 mM Tris-HCl and then disrupted by ultra-sonication, followed by centrifugation to obtain the supernatant. The recombinant proteins were purified by loading them onto a Ni-Sepharose 6FF column (GE Healthcare, USA). His-tagged target proteins were eluted with imidazole at concentrations ranging from 50 to 300 mM in 20 mM sodium phosphate buffer. The purity and molecular weight of R-DsLCYB were determined using 7% (W V1) sodium dodecyl sulfate-polyacrylamide gel electrophoresis (SDS-PAGE), with the protein bands visualized by staining with Coomassie Brilliant Blue R-250.

### Construction of Transgenic Algae

Because *D. salina* cells are sensitive to chloramphenicol, the modified pGreen0029-Cmr vector was used as the D. salina transformation vector [26]. To obtain pGreen-0029-Cmr-DsLCYB-overexpressing strains, the full-length CDS of *DsLCYB* was amplified by PCR (primers shown in Table S2) and then inserted into the modified pGreen0029-Cmr vector. *D. salina* transformation was performed according to the Agrobacterium-mediated transformation method of Claudia *et al*. [32], with slight modifications [26].

(1) The liquid algae cells in the log phase were collected and cultured for 7~10 days in DeWalne’s medium with a final concentration of 0.5 M NaCl and 5% agar until the algae grew well. This culture was used for the later *Pseudomonas* transformation experiments.

(2) The obtained vector pGreen-0029-Cmr-DsLCYB-oe and empty vector pGreen-0029-Cmr were transformed into *Pseudomonas* GV3101 according to the transformation instructions. A single colony of *Pseudomonas* was then re-inoculated into 5 ml of fresh Luria-Bertani (LB) resistant (rifampicin + chloramphenicol + gentamicin) medium, and shaken at 28 ° to OD_600_≈0.5.

(3) 5 μl of acetosyringone (100 mg/ml) was added to 5 ml of *Pseudomonas* culture solution in step (2), and cultured at 28°C for 3~4 h.

(4) 500 μl of the bacterial solution in the above step (3) was drawn and spread evenly on the algal plate in step (1), and cultivated in the dark at 25°C for 2 days.

(5) Step (4); the culture plate was washed with fresh medium containing chloramphenicol (400 μg/ml) and cefotaxime (100 μg/ml), with the algal fluid then collected by centrifugation at 5,000 ×*g* for 3 min. The algal cells were resuspended again in a fresh medium containing the same resistance concentration, and finally, the resistance screening was performed again on the solid plate for 7~10 days. The single algal colonies were picked for screening and expanded culture.

### Measurement of Total Carotenoid and Chlorophyll Content

The total carotenoid and chlorophyll contents were monitored at regular intervals by measuring optical density at OD_654_ in 80% (v/v) acetone using a UV-visible spectrophotometer (UV 2550, Shimadzu, Japan) [33]. An algal liquid OD_654_≈0.3 under RL conditions was taken as the initial sample collection time point, and then a certain volume of algal cells was collected under the three light conditions, followed by the addition 80% acetone solution and resuspension of the algal cells by vortex shaking. The extraction was carried out in the dark for about 30 min until the algal cells were completely decolorized to an off-white color, followed by centrifugation to draw off the supernatant and dilution of the sample with 80% acetone. The absorbance values were measured using a UV spectrophotometer at 470, 647, and 663 nm, with 80% acetone used as the blank control. Finally, the data were processed according to the reported formulas for carotenoids and photosynthetic pigments [34].

Ca=12.21×A663−2.81×A646 (1)

Cb=20.13×A646−5.03×A663 (2)

$$Car=\frac{1000\times A470-3.27\times Ca-104\times Cb}{229}$$ (3)

$$\text{unit pigment content = }\frac{Cx\times V\times Dilution\;factor}{FW}$$ (4)

In formula: Cx-Pigment concentration/mg·L^-1^; V- Extract volume/ml; FW- fresh weight/g.

### Carotenoid Components in the *DsLCYB* Transplastomic and Wild-Type Strain

To examine the effect of *DsLCYB* overexpression on the distribution of carotenoid components in *D. salina*, the algae cells with significant accumulation of carotenoids and a total amount that reached a maximum OD_654_≈0.6 were collected. All samples were frozen immediately in liquid nitrogen and stored at -80°C. The carotenoid composition (α-carotene, lutein, β-carotene, β-cryptoxanthin, zeaxanthin, and violaxanthin) was measured by high-performance liquid chromatography (HPLC) [35-37]. For HPLC analysis, the collected dry sample was dissolved in 200 μl of acetonitrile: methanol (20:80) solution containing 0.1% BHT. A 20 μl aliquot was then injected and separated on a YMC carotenoid S-3 um (150*4.6 mM) column at 40°C and a flow rate of 1 ml/min, with MeOH as mobile phase A and MeOH/MTBE/H2O (20: 75: 5, v/v) as mobile phase B.

### Cell Morphology, Growing Analysis in the *DsLCYB* Transplastomic and Wild-Type Strain

The cell morphology of the wild-type and DsLCYB-oe1-transformed strain was observed by light microscopy (Leica, Germany). Finally, we studied the effect of the DsLCYB-oe1 transformant on the growth of *D. salina*. The initial number of algal cells in each experimental group was the same, with culture growth monitored at regular intervals under different light conditions for 14 days by measuring the optical density at OD_654_ using a UV-visible spectrophotometer (UV 2550, Shimadzu).

### Statistical Analysis

The obtained experimental data were plotted with GraphPad Prism7 software. Then, statistical analysis was performed using Student’s *t*-test in a two-tailed analysis. *p*-values of <0.05 (*), <0.01 (**) and <0.001 (***) were considered to be statistically significant.

## Results

### Sequence Analysis and Relative Expression of *DsLCYB* in the *D. salina* Wild-Type Strain and Transformants

A 1,794 bp, full-length cDNA of LCYB from *D. salina* was isolated by PCR amplification technology. The full-length cDNA of the LCYB gene was designated *DsLCYB*. The ORF encoded a polypeptide of 597 amino acid residues with a predicted relative molecular mass of 65.69 kDa (Fig. S1). The analysis of the conserved domains showed that the *DsLCYB* protein contained the typical domains of the LCYB protein family, such as a dinucleotide-binding signature, an LCY-specific motif, cyclase motifs I and II, a charged region, two predicted TM helices, and three β-LCY CAD regions (catalytic activity domain) (Fig. S2). Phylogenetic analysis suggested that higher plants and eukaryotic green algae share a common ancestor and that *DsLCYB* clustered with green algae (Fig. S3A). To further study the conserved domains, MEME [31] was used to identify the motifs of 31 LCYB proteins, with the top 10 conserved motifs then screened out. Three motifs, motif 1, motif 3, and motif 7- were annotated as the NADB domain and cyclase motifs (Fig. S3B). The LCYBs from the same clade usually have similar conserved domains or motif compositions, indicating that the functions of these proteins were similar.

**Fig. 1:**
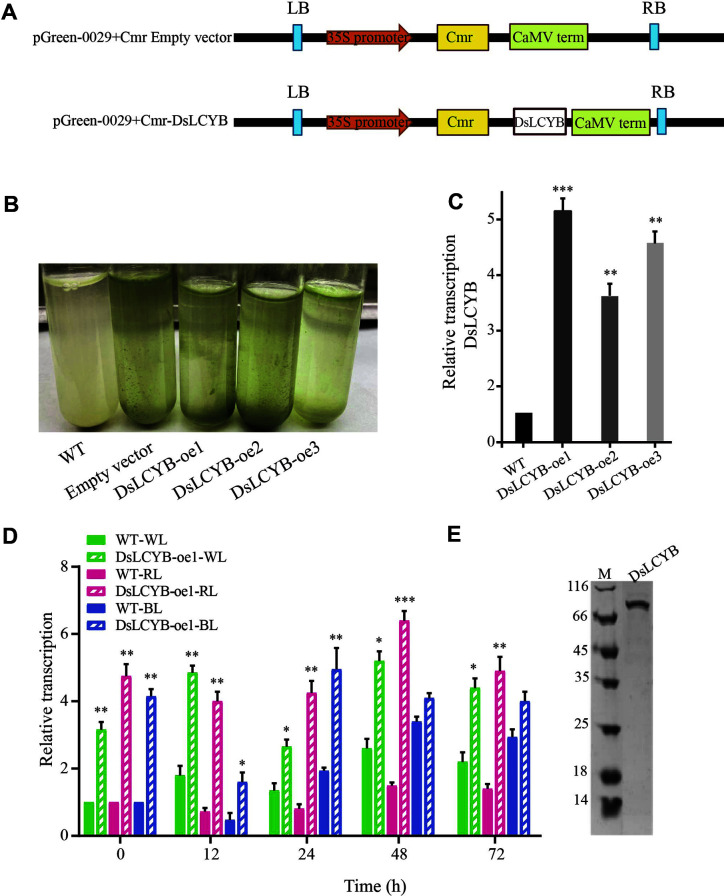
Relative transcription level analysis and heterologous expression of *DsLCYB* (**A**) Vector maps of pGreen-0029-Cmr and pGreen-0029-Cmr-DsLCYB constructed for overexpression in *D. salina*. (**B**) Screening of the wild-type and transformed lines for resistance using chloramphenicol (400 μg/ml). (**C**) Gene expression level of *DsLCYB* in the wild-type and *DsLCYB* transplastomic under the same physiological conditions. (**D**) Gene expression level of *DsLCYB* in the wild-type and overexpressed strain was measured by RT-qPCR under different lighting conditions, with the β- tubulin gene was used as an internal reference. (**E**) The SDS-PAGE of purified *DsLCYB* recombinant protein. The arrow pointed the target protein band; Lane M, protein marker (kDa). WL: white light, RL: red light, BL: blue light. The significant difference between the two groups was obtained by the student's test. Error bars indicate standard error. *p* values of <0.05 (*), <0.01 (**), and <0.001 (***) were considered to be significant statistically.

The *DsLCYB* gene fragment was successfully inserted into the linearized vector pGreen-0029-Cmr (Fig. 1A), with three overexpression strains (DsLCYB-oe1, DsLCYB-oe2, DsLCYB-oe3) obtained after screening for 7~10 days with chloramphenicol at a concentration of 400 μg/ml (Fig. 1B). We next quantified and compared the expression levels of *DsLCYB* in the *D. salina* transformants with that of the wild-type strain, in which the transcription level of *DsLCYB* was 1, and which was also used as the negative control. Meanwhile, the DsLCYB-oe1, DsLCYB-oe2, and DsLCYB-oe3 transformants showed significant increases in LCYB transcript levels, reaching 5.2-, 3.5-, and 4.4-fold higher than that of the wild-type strain, respectively (Fig. 1C). For the following analyses, we selected the DsLCYB-oe1 transformant that grew rapidly at higher antibiotic concentrations and with the best transformation effect. The regularity of the response to *DsLCYB* of the wild type and overexpressing lines under different light stress was determined using RT-qPCR. The results showed that the transcript level of *DsLCYB* was higher in the DsLCYB-oe1 transformant strain than that of the wild-type strain under different lighting stress. The expression of *DsLCYB* was induced strongly by WL, and RL, and peaked at 48 h (2.22-fold, and 5.50-fold, respectively). However, there was no significant change for 48 h under BL conditions. It was presumed that this time had the greatest impact on carotenoid synthesis (Fig. 1D). To further characterize the catalytic properties of *DsLCYB*, the gene was heterologously expressed in *E. coli* BL21 (DE3). *E. coli* BL21 (DE3) containing *DsLCYB* recombinant plasmid was induced by IPTG overnight, broken by ultrasound, and purified by Ni-NTA affinity chromatography to obtain the purified recombinant protein *DsLCYB*. The results of SDS-PAGE detection showed that the positions of the purified recombinant protein band were consistent with the predicted molecular weight of the fusion protein (the size of the fusion tag was about 20 kDa), and that the protein band was single, with no obvious foreign bands around, and a purity that met the requirements of subsequent experiments (Fig. 1E).

### Carotenoid Content and Components of *D. salina* Wild-Type Strain and Transformants under Different Light Conditions

**Fig. 2:**
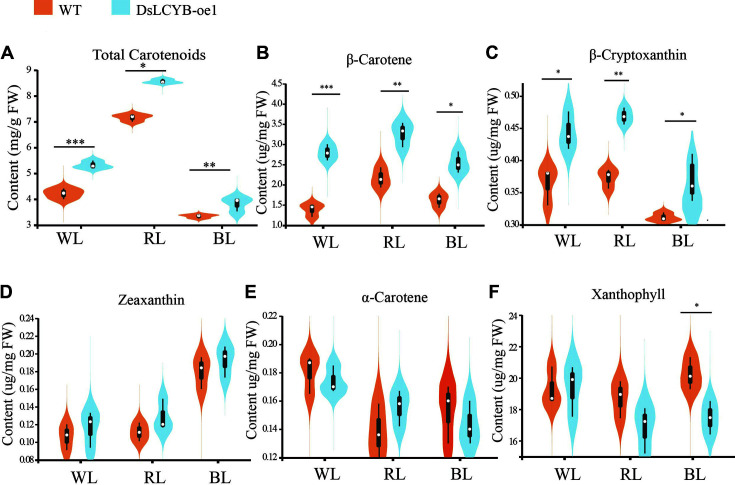
The content of total carotenoids and components in *D. salina* were measured by spectrophotometer and HPLC respectively. (**A**) Content of total carotenoids. (**B**) β-carotene, (**C**) β-cryptoxanthin, (**D**) zeaxanthin, (**E**) α- carotene, and (**F**) xanthophyll. WL: white light, RL: red light, BL: blue light. The significant difference between the two groups was obtained by the student's test. Error bars indicate standard error. *p* values of <0.05 (*), <0.01 (**), and <0.001 (***) were considered to be significant statistically.

We successfully obtained a DsLCYB-oe1 transgenic strain to determine whether *DsLCYB* promoted the accumulation of β-carotene and affected the proportional distribution of the main components. First, we measured carotenoid production in the wild-type strain and DsLCYB-oe1 transformant within 72 h of light stress and showed that the accumulation of carotenoids reached a peak at 48 h (Fig. S4). In the wild-type strain, the highest carotenoid was produced at about 4.22, 7.14, and 3.48 mg/g of light culture, respectively. In comparison, the DsLCYB-oe1 transformant produced carotenoid at 5.16, 8.46, and 3.92 mg/g of culture under the same conditions, respectively. Therefore, the total carotenoid production in the transformant tended to be higher than that in the wild-type strain (up to 1.22-, 1.21-, and 1.12-fold higher, respectively). Moreover, compared with WL and BL stresses, the DsLCYB-oe1 transformant significantly increased total carotenoid production (1.67- and 2.2-fold, respectively) under RL (Fig. 2A). On the other hand, carotenoid composition measured by HPLC showed that the contents of β-carotene and β-cryptoxanthin in the DsLCYB-oe1 transformant were increased significantly compared with those in the wild type (2.5-3.3 μg/g (1.5-1.8 fold) and 0.36-0.47 μg/g (1.1-1.3 fold), respectively Figs. 2B, 2C). However, there were no significant changes in the content of zeaxanthin and lutein (Figs. 2D and 2E). In addition, xanthophyll only decreased significantly under BL conditions (Fig. 2F). These results showed that overexpression of *DsLCYB* promoted the accumulation of carotenoids in *D. salina*.

### Expression Analysis of Carotenoid Biosynthesis Pathway and Responsive Genes

**Fig. 3:**
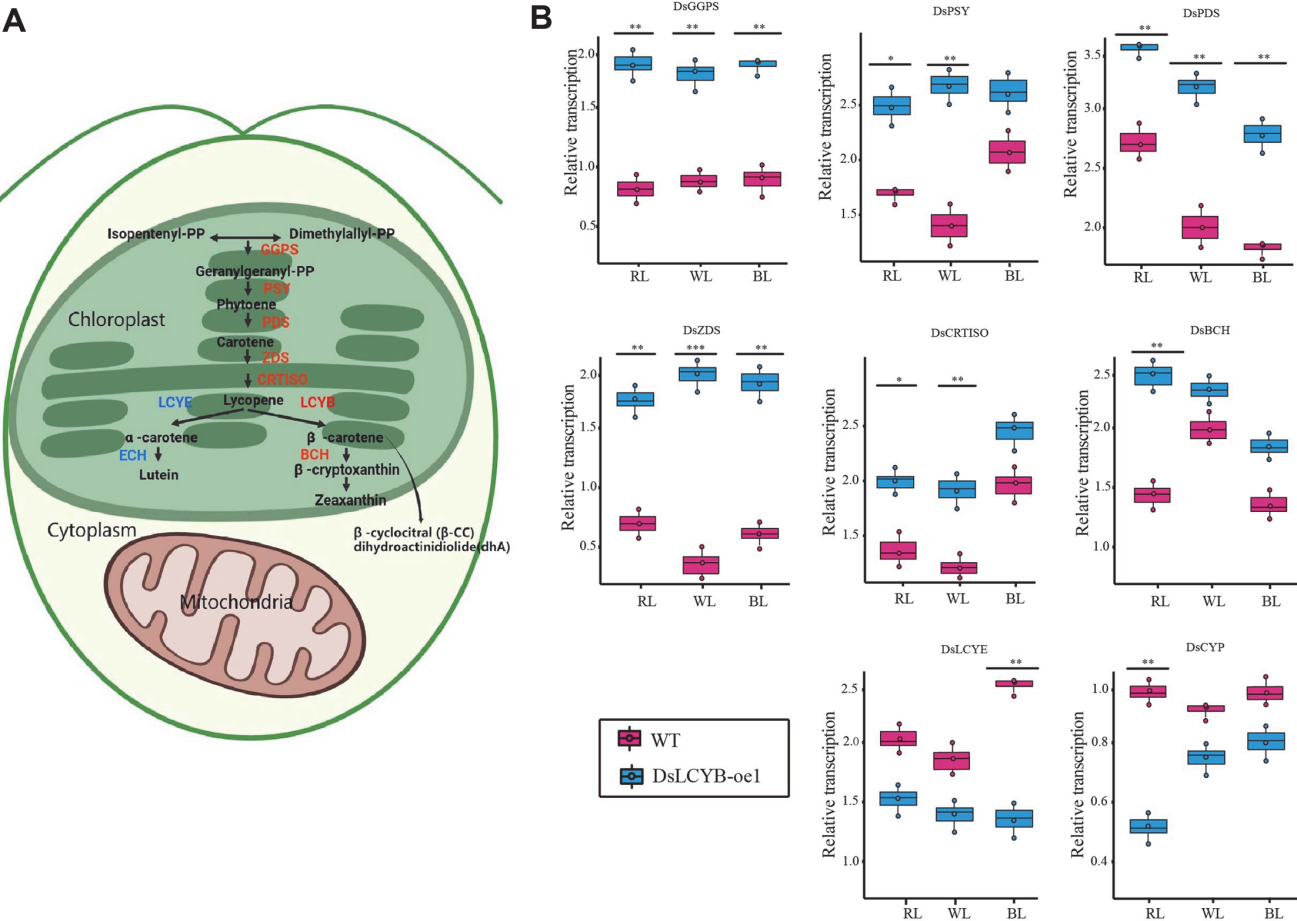
Brief schematic of the carotenoid biosynthesis pathway in algae and transcriptional levels of genes involved in the carotenoid biosynthesis in DsLCYB-oe1 over-expressing transformant under different lighting conditions. Gene expression level of *DsGGPS*; *DsPSY*; *DsPDS*; *DsZDS*; *DsCRTISO*; *DsBCH*; *DsLCYE*; *DsCYP* were measured by RT-qPCR, and β-tubulin was set as control. WL: white light, RL: red light, BL: blue light. The significant difference between the two groups was obtained by the student's test. Error bars indicate standard error. *p* values of <0.05 (*), <0.01 (**), and <0.001 (***) were considered to be significant statistically. GGPS, geranylgeranyl pyrophosphate synthase; PSY, phytoene synthase; PDS, phytoene desaturase; ZDS, ζ-carotene desaturase; CRTISO, carotene isomerase; BCH, β-carotene hydroxylase; LCYB, lycopene β-cyclase; LCYE, lycopene ε-cyclase; CYP, cytochrome P450. Genes are marked up-regulated in red and downregulated are marked in blue.

To investigate the regulatory mechanisms of carotenoid biosynthesis, the expression levels of the upstream genes of carotenoid synthesis (*DsGPPS*, *DsPSY*, *DsPDS*, *DsZDS*, *DsCRTISO*), lycopene cyclase (*DsLCYB*, *DsLCYE*), α-carotenoid downstream gene (*DsCYP*), and β-carotene downstream gene (*DsBCH*) were detected by RT-qPCR. *DsGGPS*, *DsPDS*, and *DsZDS* were upregulated significantly in the transgenic *D. salina* overexpressing DsLCYB-oe1 under different light conditions. *DsPSY* and *DsCRTISO* were induced to become upregulated under WL and RL stresses. Regarding the two enzymes involved in the cyclization of lycopene, *DsLCYB* was upregulated significantly in the DsLCYB-oe1 transformant (Fig. 1A), whereas *DsLCYE* was downregulated significantly at the same time. These changes affected the metabolic flow of carotenoids and the accumulation of downstream products. The downstream of carotenoid genes encoding *DsBCH* and *DsCYP* were upregulated significantly in the transgenic line only under RL compared to that observed in control plants (Figs. 3A and B).

### Growth and Photosynthesis Analysis in the *DsLCYB* Transplastomic

Previous results showed that carotenoids accumulated abundantly in the DsLCYB-oe1 overexpressor, and it was hypothesized that the increase in plant biomass may be due to these additional disturbances that are related directly to the structure-function in the photosynthetic apparatus. Therefore, we evaluated the cell morphology and growth parameters in these lines.

**Fig. 4:**
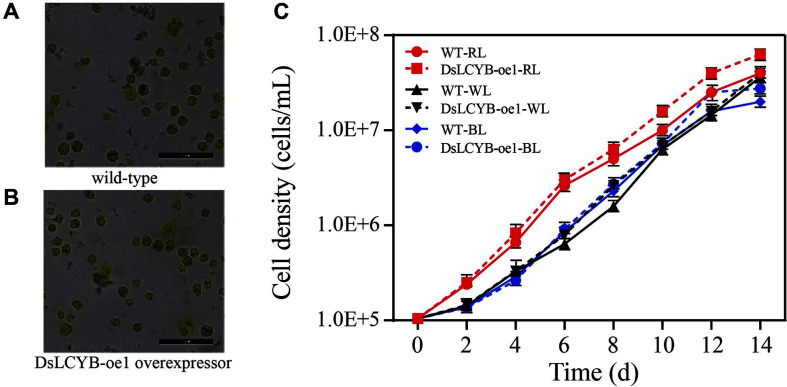
Morphology and growth analysis of *D. salina*. The morphology of wild-type and transformant was shown by electron microscopy images of algal cells in the logarithmic phase (A, B). Bars = 100 μm. (A) Cell morphology of wild-type *D. salina* (B) Cell morphology of DsLCYB-oe1 overexpressor (C) Growth curves of wild-type and transformant under different light culture conditions using a Neubauer hemocytometer for 14 d. WL: white light, RL: red light, BL: blue light.

First, cell morphology was observed by light microscopy in the wild-type and transformed algae. Compared with the wild type, no changes in the morphological structure of the cells were observed in the overexpression system (Figs. 4A, 4B). In contrast, cell debris was present in the transformation system, indicating that some algal cells were broken after transformation without affecting cell morphology, thereby not affecting the growth of living algal cells (Fig. 4B).

We then investigated the effect of the DsLCYB-oe1 transformant on the growth of *D. salina*. The initial number of algal cells in each experimental group was the same, and the growth curves of the different algal strains were then monitored under different light conditions for 14 days. Growth was measured in terms of cell numbers using a Neubauer hemocytometer. In the wild-type strain culture, the algal cell density increased from the initial density of 1 × 10^5^ cells/ml to 7 × 10^6^ cells/ml 12 days post-inoculation. However, in the DsLCYB-oe1 culture, algal cell density reached a plateau at 14 days post-inoculation at a cell density of 7 × 10^6^ cells/ml (Fig. 4C).

**Fig. 5:**
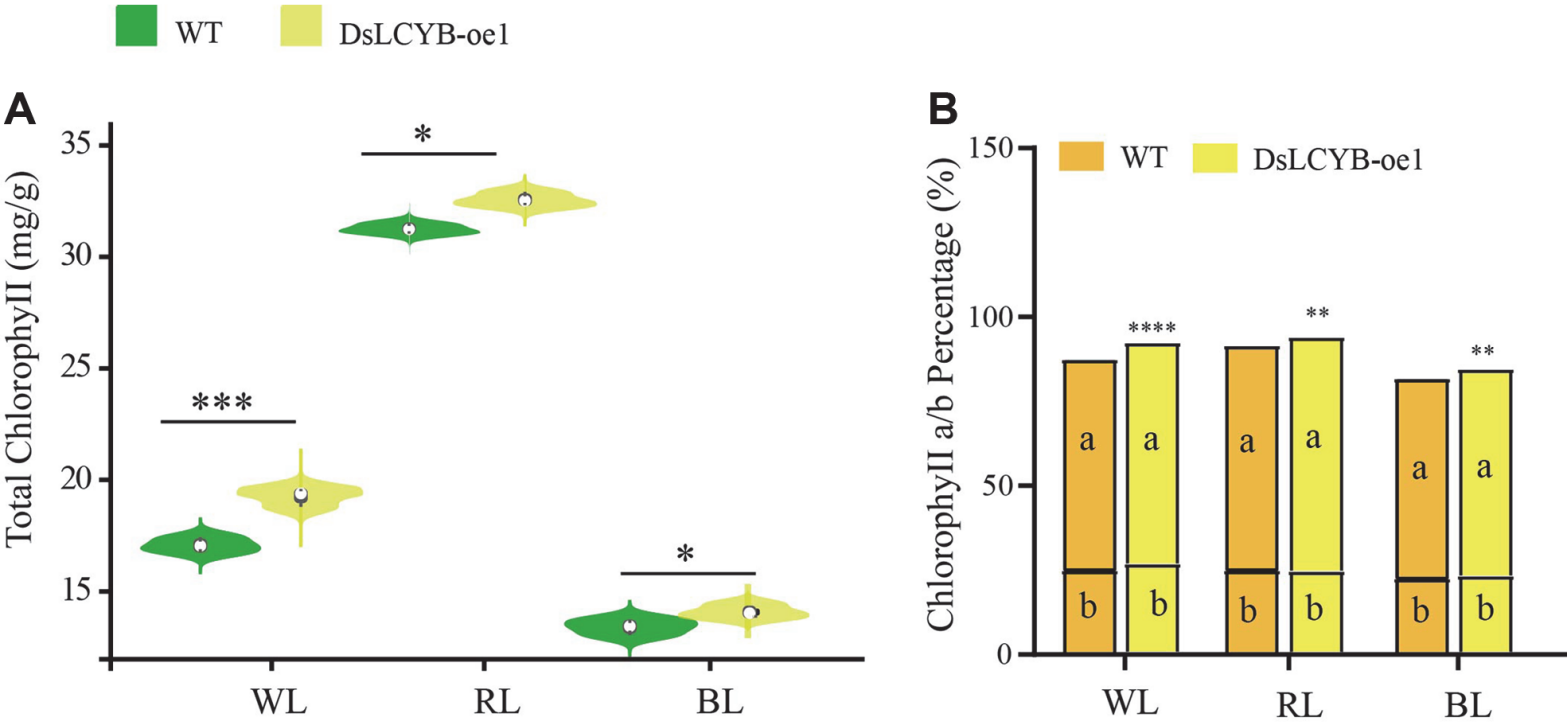
Content chlorophyll of *D. salina* in the wild-type and overexpression strain were measured by spectrophotometer at 48 h under different lighting conditions. (**A**) The total chlorophyll content. (**B**) The chlorophyll a/b content percentage of total chlorophyll content. WL: white light, RL: red light, BL: blue light. The significant difference between the two groups was obtained by the student's test. Error bars indicate standard error. *p* values of <0.05 (*), <0.01 (**), and <0.001 (***) were considered to be significant statistically.

Finally, the results showed that the contents of total chlorophyll, chlorophyll a, and chlorophyll b reached the highest at 48 h (Fig. S5). The content of total chlorophyll in the wild-type and transformed line under RL was significantly higher (32.5 mg/g) than that observed under WL (19.2 mg/g) and BL (14.4 mg/g) conditions. Meanwhile, the data analysis showed that the chlorophyll content of the DsLCYB-oe1 transformed line followed an increasing trend, which was 1.25-1.28, 1.07-1.12, and 1.09-1.11-fold higher than that of control plants under light stress treatments, respectively. The percentage content of chlorophyll and chlorophyll a/b at 48 h was increased significantly in the DsLCYB-oe1 transformant compared with that in the wild type (Figs. 5A and 5B).

## Discussion

β-Carotene, a precursor of vitamin A, has important physiological functions and effectively prevents damage caused by oxidative stress, methotrexate-induced oxidative damage, and apoptosis [38, 39]. An ideal model organism, *D. salina*, has been shown to accumulate a large amount of β-carotene under stress caused by environmental factors [18-20]. The efficient production of β-carotene by microalgae is a promising breeding goal. Understanding the regulatory mechanisms of β-carotene metabolism and biosynthesis will allow researchers to develop more efficient ways to increase β-carotene content for industrial production applications.

The regulation of *DsLCYB* on metabolic flux distribution of carotenoid biosynthesis is directional. In photosynthetic organisms, the biosynthetic pathway of carotenoids is divided into two main metabolic branches regulated by LCYE and LCYB, which produce α- and β-carotene, respectively [12, 40]. In the current study, the overexpression of the *DsLCYB* gene in transgenic algae enhanced the conversion of lycopene to the β-carotene metabolic branch, thereby increasing the yields of β-carotene and β-cryptoxanthin in the transformants. Significantly, the content of the important product, xanthophyll, in the α-branch, was found to be insignificantly reduced in DsLCYB-oe1 algae under BL conditions, while there was no significant change in α-carotenoid content. In contrast, researchers have reported that key lycopene cyclases (α and β cyclases) in the carotenoid synthesis pathway altered carbon flow in the branch of α- and β-carotene in higher photosynthetic plants [41]. It has also been reported that LCYB is transformed in some photosynthetic organisms (*wheat* [42], *Daucus carota* [15], *sweet potato* [14], and *tobacco* [43]), with overexpression improving carotenoid content (α-carotene, β-carotene, lutein, β-cryptoxanthin, zeaxanthin, violaxanthin, and neoxanthin), photosynthetic efficiency, and abiotic stress tolerance. The directional regulation of the metabolic influx to α- or β-carotene was also demonstrated in other lower microalgae. The overexpression of the LCYE gene in *Chlamydomonas reinhardtii* increased the content of lutein but did not inhibit the production of β-carotene [44]. These results indicated that the regulation of the carotenoid metabolic cyclases (α and β cyclases) in lower microalgae can be orientated towards accumulation of α- or β-branched metabolites, and that microalgae are beneficial for the targeted and rapid production of specific carotenoid products. Furthermore, the upregulation of the *DsLCYB* gene accelerated metabolic flux to the β-carotene metabolic branch in the transformants. This resulted in upregulation of upstream and downstream genes involved in carotenoid synthesis, with the exception of *DsCYP*. Therefore, we showed that β-carotene and β-cryptoxanthin content increased significantly (1.13-2.41-fold, 1.12-1.38-fold, respectively)(Figs. 2B and 2C), which was consistent with accelerated carotenoid biosynthesis and accumulation of additional carotenoids.

The photosynthetic efficiency of photosynthetic organisms affects the carotenoid synthesis. A study by Ryu *et al*. [45] showed that carotenoid biosynthesis in cyanobacteria was related to photosynthesis. Research carried out by Busch *et al*. [46] demonstrated that the chlorophyll content of tobacco leaves could be influenced by the overexpression of *NtPSY*, with the ratio of chlorophyll to carotenoids being altered. The chlorophyll-to-carotenoids mole ratio was also analyzed in the current study, and was calculated by using the average content of carotenoids and chlorophyll shown in Figs. 2A and 5A. This showed that extra chlorophyll accumulated in *D. salina*, with the mole ratio of chlorophyll to carotenoids varying significantly in the transgenic line, (3.64 in the DsLCYB-oe1 transformant, and 4.07 in the control group). This suggested that chlorophyll content, which correlates with photosynthesis, was involved in the adaption to the impact caused by overexpression. The content per algal cell was also analyzed in the current study, and was calculated by using the average content of carotenoids and cell density shown in Figs. 2A and 4C. The results showed that the content of carotenoids in each algal cell varied significantly between the transgenic line and wild type (0.89 pg/cell in DsLCYB-oe1 overexpressor, and 0.62 pg/cell in the control group) at 48 h under red stress. This suggested that carotenoid production increased due to an increase in unit cell yield.

In conclusion, *DsLCYB* was cloned and analyzed for functional characterization from *D. salina*. Our results showed that its overexpression significantly increased carotenoid content and improved photosynthesis in *D. salina*. Significantly, overexpression of *DsLCYB* promoted the flow of carotenoid metabolism to the β-branch of the pathway leading to accumulation of β-carotene and β-cryptoxanthin, thereby changing the ratio of carotenoid components without having negative effects on their growth and lutein content. These studies provide a basis for regulating the directional changes of carotenoid synthesis and carotenoid composition ratio at the transcriptional level and accordingly have major industrial significance.

### Relative Transcription Level Analysis and Heterologous Expression of *DsLCYB*

(A) Vector maps of pGreen-0029-Cmr and pGreen-0029-Cmr-DsLCYB constructed for overexpression in *D. salina*. (B) Screening of the wild-type and transformed lines for resistance using chloramphenicol (400 μg/ml). (C) Gene expression level of *DsLCYB* in the wild-type and *DsLCYB* transplastomic under the same physiological conditions. (D) Gene expression levels of *DsLCYB* in the wild-type and overexpressed strains were measured by RT-qPCR under different lighting conditions, with the β-tubulin gene used as an internal reference. (E) The SDS-PAGE of purified *DsLCYB* recombinant protein. The arrow indicates the target protein band; Lane M, protein marker (kDa). WL: white light, RL: red light, BL: blue light. The significant difference between the two groups was obtained by Student's *t*-test. Error bars indicate standard error. *p*-values of <0.05 (*), <0.01 (**), and < 0.001 (***) were considered to be significant statistically.

### The Content of Total Carotenoids and Components in *D. salina* were Measured by Spectrophotometer and HPLC Respectively

(A) Content of total carotenoids. (B) β-carotene, (C) β-cryptoxanthin, (D) zeaxanthin, (E) α-carotene, and (F) xanthophyll. WL: white light, RL: red light, BL: blue light. The significant difference between the two groups was obtained by the Student's *t*-test. Error bars indicate standard error. *p*-values of <0.05 (*), <0.01 (**), and <0.001 (***) were considered to be significant statistically.

### Brief Schematic of the Carotenoid Biosynthesis Pathway in Algae and Transcriptional Levels of Genes Involved in the Carotenoid Biosynthesis in DsLCYB-oe1 Overexpressing Transformant under Different Lighting Conditions

Gene expression levels of *DsGGPS*; *DsPSY*; *DsPDS*; *DsZDS*; *DsCRTISO*; *DsBCH*; *DsLCYE*; *DsCYP* were measured by RT-qPCR, and β-tubulin was set as control. WL: white light, RL: red light, BL: blue light. The significant difference between the two groups was obtained by the Student's *t*-test. Error bars indicate standard error. *p*-values of <0.05 (*), <0.01 (**), and <0.001 (***) were considered to be significant statistically. GGPS, geranylgeranyl pyrophosphate synthase; PSY, phytoene synthase; PDS, phytoene desaturase; ZDS, ζ-carotene desaturase; CRTISO, carotene isomerase; BCH, β-carotene hydroxylase; LCYB, lycopene β-cyclase; LCYE, lycopene ε-cyclase; CYP, cytochrome P450. Genes are marked upregulated in red and downregulated are marked in blue.

### Morphology and Growth Analysis of *D. salina*

The morphology of wild type and transformant was shown by electron microscopy images of algal cells in the logarithmic phase (A, B). Bars = 100 μm. (A) Cell morphology of wild-type *D. salina* (B) Cell morphology of DsLCYB-oe1 overexpressor (C) Growth curves of wild type and transformant under different light culture conditions using a Neubauer hemocytometer for 14 days. WL: white light, RL: red light, BL: blue light.

### Content Chlorophyll of *D. salina* in the Wild-type and Overexpression Strain Were Measured by Spectrophotometer at 48 h under Different Lighting Conditions

(A) The total chlorophyll content. (B) The chlorophyll a/b content percentage of total chlorophyll content. WL: white light, RL: red light, BL: blue light. The significant difference between the two groups was obtained by the Student's *t*-test. Error bars indicate standard error. *p*-values of <0.05 (*), <0.01 (**), and <0.001 (***) were considered to be significant statistically.

## Supplemental Materials

Supplementary data for this paper are available on-line only at http://jmb.or.kr.
