# Supplementary material for: DsLCYB Directionally Modulated β-Carotene of the Green Alga Dunaliella salina under Red Light Stress
Source: J Microbiol Biotechnol. 2022 Oct 31;32(12):1622–31. doi: 10.4014/jmb.2208.08044 (PMC9843872; doi:10.4014/jmb.2208.08044)
Supplement: Supplementary file 1 [file jmb-32-12-1622-supple.pdf]

J. Microbiol. Biotechnol.
https://doi.org/10.4014/jmb.2208.08044

```
          10         20         30         40         50         60
1   mqqmlssrss nvlscnpqll psqhaysiss arictetpqs sqetlgwrpl svthsathgk
          70         80         90        100        110        120
61  arrtraqall qqkpetpyfs ppapsvtgpv qyyyrepsaw ptqqdvqvvy hdlqsqptad
         130        140        150        160        170        180
121 llvvgsgpsg lavaervaag gfsvcvidld pyapmipnyg vwvdemqamg leeclevvwp
         190        200        210        220        230        240
181 qakvwldndk sgerylkray grmdrpmlkk lllqkctsng vtfltskvag vshgggtstv
         250        260        270        280        290        300
241 tlsdgrtmqg tmvldatgha cklvnfdqkf dpgyqgaygi taeveshpfe ldtmlfmdwr
         310        320        330        340        350        360
301 dehthsnpam rasnealptf lyvmpytknk vfleetslva rpamgfdelk erlkariewl
         370        380        390        400        410        420
361 gikvtkvede eyclipmggv lpqhpqrvlg iggtagmvhp stgfmmtrml gsapvvadai
         430        440        450        460        470        480
421 idqlsrptdk atdagaarql vteqeaeema aavwratwpv erirqraffs fgmemlltln
         490        500        510        520        530        540
481 lqqtrdffaa ffslsdfqwq gflsarlsft qlisfglslf skatsatrtn llrlgipglv
         550        560        570        580        590        600
541 qmllvllptv tgyykedktv mdkkfahdqa aaaaaktakq qespsaspvs qggnpga
```

Fig. S1 Amino acid sequence of DsLCYB

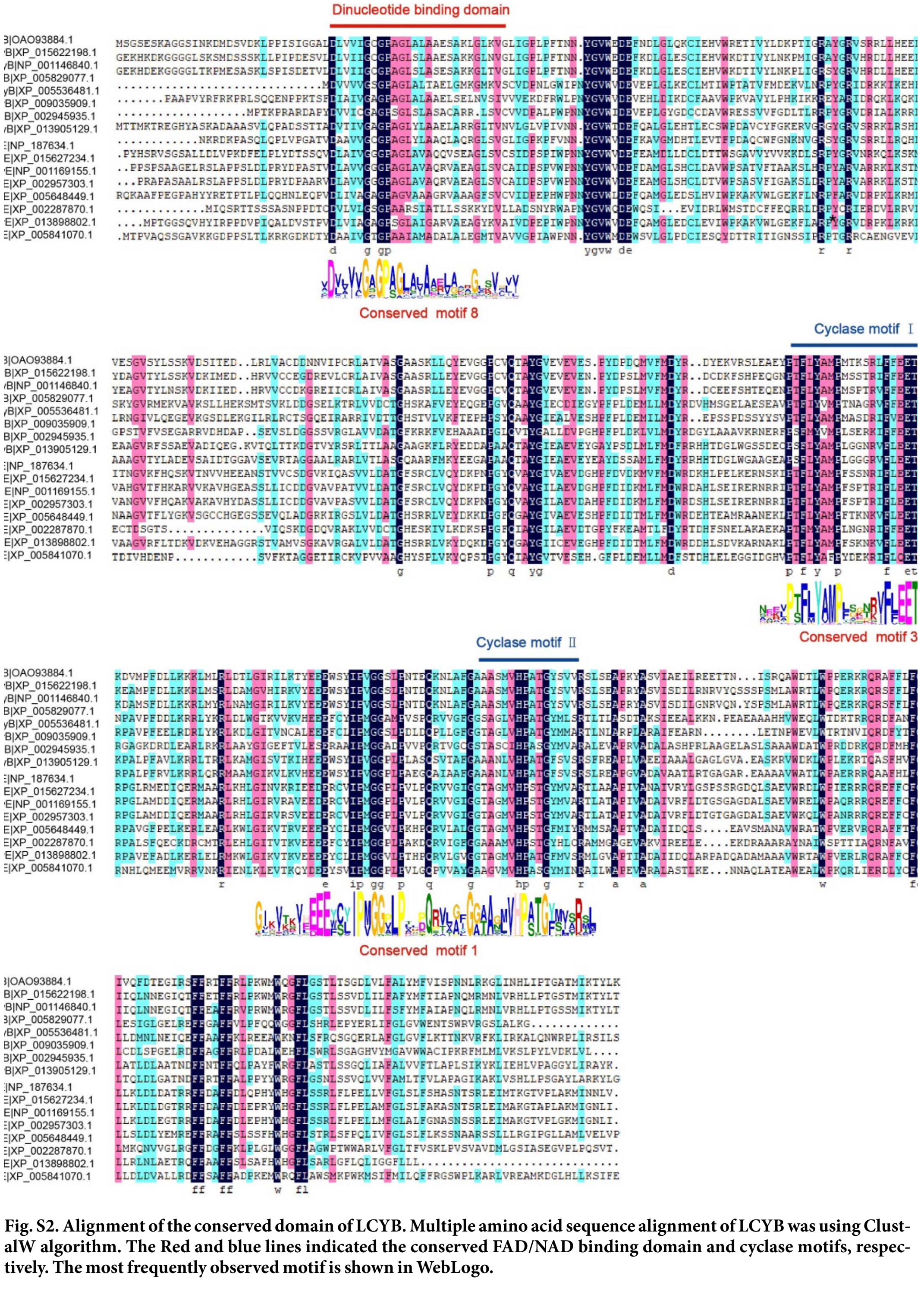

Fig. S2. Alignment of the conserved domain of LCYB. Multiple amino acid sequence alignment of LCYB was using ClustalW algorithm. The Red and blue lines indicated the conserved FAD/NAD binding domain and cyclase motifs, respectively. The most frequently observed motif is shown in WebLogo.

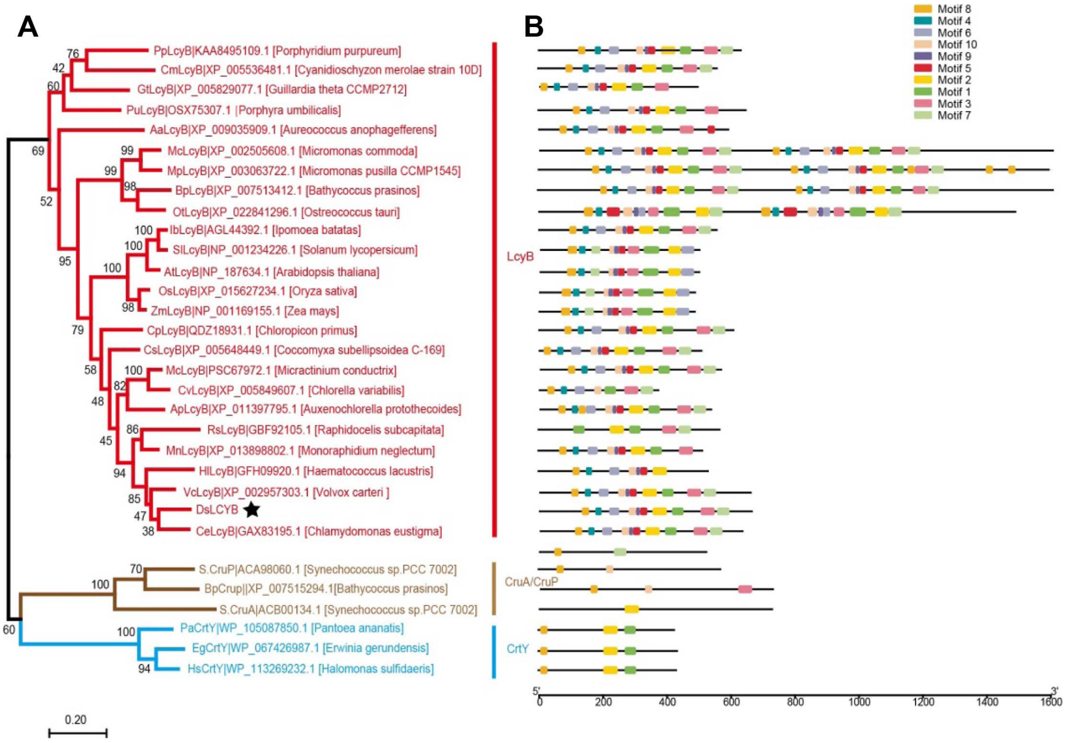

Fig. S3. Phylogenetic tree and motif compositions of lycopene cyclases from cyanobacteria, algae, and angiosperms. (A) The phylogenetic tree was generated by using NJ method of MEGA7 program. (B) The motifs of 1-10 were shown in boxes of different colors. The black lines indicate non-conserved sequences, and the black pentagram is the LCYB protein of *D. salina*.

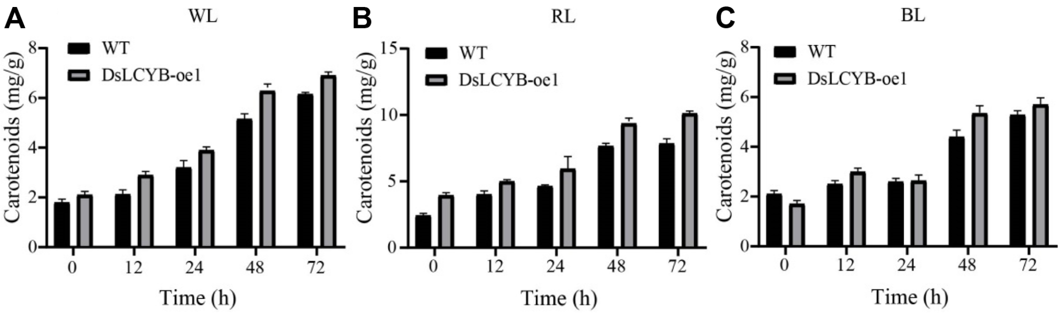

Fig. S4. Total carotenoid content of *D. salina* was measured by spectrophotometer in wild-type and overexpression strain under different lighting conditions was extracted by acetone method. WL: white light, RL: red light, BL: blue light.

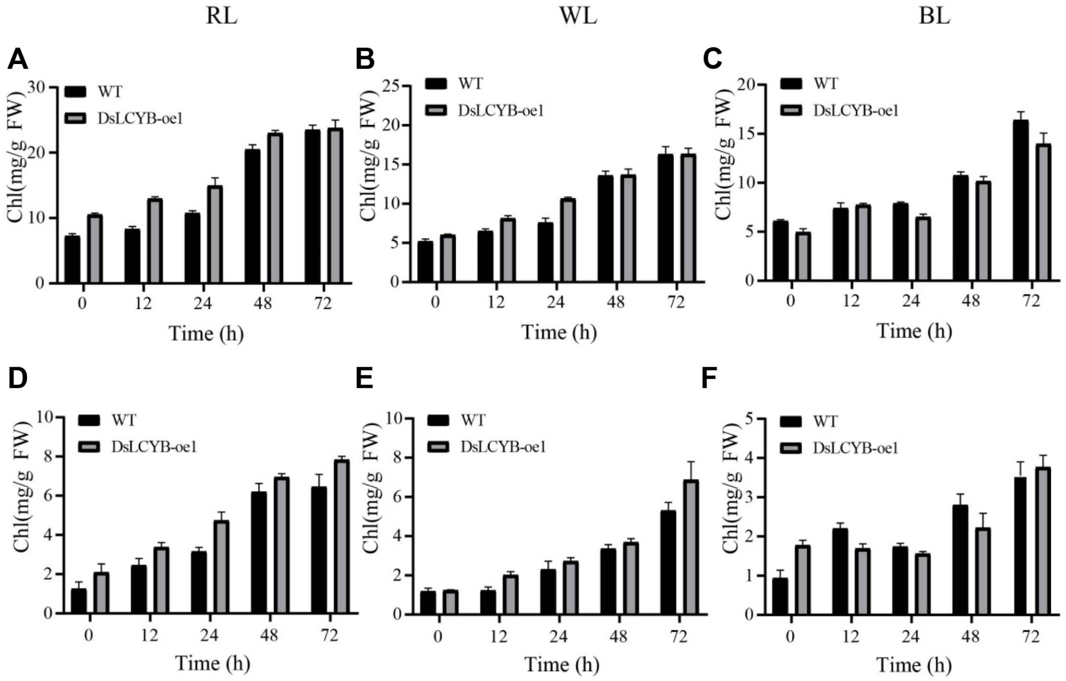

Fig. S5. Contents chlorophyll a and chlorophyll b of *D. salina* were measured by spectrophotometer in wild-type and over-expression strain. (A-C) The content of chlorophyll a under light stress (D-F) The content of chlorophyll b under light stress. WL: white light, RL: red light, BL: blue light.

Table S1. Primers of qRT-PCR

Gene name	Foward primer（5'→3'）	Reverse primer（5'→3'）
DsGGPS	AAGTGCCACGAAGACCCG	ATGTAGCCCTGGAAATCAAA
DsPSY	CTCATGACACCAGTGCAGGC	CCGCGTCCAGCACATCATAG
DsPDS	GAGACTGGTATGAGACCGGCC	GGCAGGGATGTCTGGGAACT
DsZDS	CGCTACAATGGTTGGGTGACG	GCAGGAGAAGAACGCATCAGG
DsCRTISO	GGACATGTGCTGCAGGCTATT	GGACAACACGGAGCCAAAGTT
DsLCYB	GCGTATGGGCGAATGGAT	ATGCGACTCAACCTCTGCTGT
DsBCH	GACATCAAGTGGGCATACAA	ACGCCAGTGAAGAACAGC
DsECH	TTCAAGTGGTCCAAACGCTGTA	AAGGCGATGTTGCGAGGC
DsLCYE	AGAGCTTGGCCTCCACTCTC	GCCTCATCAATCCACACACC
β-tubulin	GTGGAGAACGCCGATGAGT	AGCAGGTGACACCGGACAT

Table S2. Primers for transient plant expression vector of DsLCYB

Gene name	Primer（5'→3'）
DsLCYBF	GGATCC ATGCAACAAATGCTCAGCAGTCGAA
DsLCYBR	TCTAGA TCAAGCACCAGGGTTGCCACCTTGA
